# Comparison of olanzapine 2.5 mg and 5 mg in the prevention of chemotherapy-induced nausea and vomiting: a Japanese nationwide database study

**DOI:** 10.1007/s10147-024-02603-2

**Published:** 2024-08-18

**Authors:** Hiroe Suzuki-Chiba, Takaaki Konishi, Shotaro Aso, Kanako Makito, Hiroki Matsui, Taisuke Jo, Kiyohide Fushimi, Hideo Yasunaga

**Affiliations:** 1 Department of Clinical Epidemiology and Health Economics, School of Public Health, The University of Tokyo, 7-3-1 Hongo, Bunkyo-Ku, Tokyo, 113-8655 Japan; 2 Department of Real World Evidence, Graduates School of Medicine, The University of Tokyo, Tokyo, Japan; 3 Department of Biostatistics and Bioinformatics, Graduate School of Medicine, The University of Tokyo, Tokyo, Japan; 4 Department of Respiratory Medicine, The University of Tokyo, Tokyo, Japan; 5 Department of Health Services Research, The University of Tokyo, Tokyo, Japan; 6 Department of Health Policy and Informatics, Tokyo Medical and Dental University Graduate School of Medicine, Tokyo, Japan

**Keywords:** Antiemetics, Chemotherapy, Cisplatin, Lung cancer, Olanzapine

## Abstract

**Background:**

Olanzapine is prescribed as prophylaxis for chemotherapy-induced nausea and vomiting at a dose of 2.5 or 5 mg in Asian countries. We compared the effectiveness of olanzapine 2.5 mg and 5 mg in preventing chemotherapy-induced nausea and vomiting among patients receiving high-emetogenic chemotherapy for lung cancer.

**Methods:**

Using a Japanese national inpatient database, we identified patients who received olanzapine doses of 2.5 or 5 mg during high-emetogenic chemotherapy for lung cancer between January 2016 and March 2021. We conducted a 1:1 propensity score-matched analysis with adjustment for various factors, including those affecting olanzapine metabolism. The outcomes were additional antiemetic drug administration (within 2–5 days after chemotherapy initiation), length of hospital stay, and total hospitalization costs.

**Results:**

Olanzapine 2.5 and 5.0 mg were used in 2905 and 4287 patients, respectively. The propensity score-matched analysis showed that olanzapine 2.5 mg administration was significantly associated with a higher proportion of additional antiemetic drug administration (36% vs. 31%, p < 0.001) than olanzapine 5 mg. The median length of hospital stay was 8 days in both groups. Total hospitalization cost did not differ significantly between the two doses of olanzapine (5061 vs. 5160 USD, p = 0.07). The instrumental variable analysis demonstrated compatible results.

**Conclusion:**

Prophylactic use of olanzapine 2.5 mg during chemotherapy for lung cancer was associated with a higher rate of additional antiemetic drugs than olanzapine 5 mg.

**Supplementary Information:**

The online version contains supplementary material available at 10.1007/s10147-024-02603-2.

## Introduction

Chemotherapy-induced nausea and vomiting (CINV) is a major side effect of chemotherapy and affects patients' quality of life [1, 2]. CINV is common with high-emetogenic chemotherapy (HEC). HEC often requires a combination of three antiemetic drugs: a neurokinin 1 (NK1) receptor antagonist, a 5-hydroxytryptamine (5-HT3) receptor antagonist, and dexamethasone. However, half of patients receiving HEC in a previous study developed CINV due to the insufficient effectiveness of the three-drug combination [3].

Recent studies have demonstrated the effectiveness of olanzapine. Accordingly, the American Society of Clinical Oncology, National Comprehensive Cancer Network, and Multinational Association of Supportive Care in Cancer recommend the use of olanzapine as CINV prophylaxis for patients receiving HEC [4–6]. Furthermore, compared to the three-drug combination, olanzapine is cost-effective for patients with nausea and vomiting [7, 8].

Olanzapine 10 mg/day is safely used in the United States (US). However, due to the risk of oversedation, olanzapine 5 mg is used in Asian countries [9–12]. Furthermore, olanzapine 2.5 mg is prescribed for patients receiving HEC in real-world settings in Asian countries to avoid sleepiness. Previous retrospective studies have reported the effectiveness and safety of olanzapine 2.5 mg [13]. Although olanzapine 2.5 mg has not yet been approved as an antiemetic, clinicians may frequently prescribe it, considering its side effects (such as sleepiness). Specifically, several randomized controlled trials were conducted in Asian countries using olanzapine 2.5 mg [14, 15]. However, these previous studies had limitations, such as small sample sizes (n = 112 and 275) and lack of investigation of factors that influence olanzapine metabolism, such as smoking and drugs that induce cytochrome P450 enzymes [16–20]. A recent study has shown the effectiveness of olanzapine through subgroup analysis of risk factors for CINV [21]. However, no study has investigated factors affecting olanzapine metabolism. Additionally, previous studies of olanzapine 5 mg had a small proportion of patients aged ≥ 75 years. Therefore, the effectiveness and safety of olanzapine in the elderly remain unclear [9].

The optimal dose of olanzapine used as CINV prophylaxis remains unclear in Asian patients, particularly in the elderly. Therefore, we compared the effectiveness of two olanzapine doses (2.5 and 5 mg), using a nationwide inpatient database in Japan.

## Patients and methods

### Data source

This retrospective cohort study used patient data from the Diagnosis Procedure Combination database. As of November 2022, this nationwide database contained hospital administrative claims data and discharge abstracts of approximately 8,000,000 inpatients from more than 1200 acute care hospitals[22]. Participation in this database is compulsory for academic hospitals and voluntary for community hospitals. The database contains the following information: patient’s age, sex, body height, body mass index (BMI), smoking history, primary diagnosis, comorbidities at admission (International Classification of Diseases [ICD]-10 code), prescription information, tumor–node–metastasis stage of malignant tumor, length of hospital stay, discharge status, activities of daily living (ADL) following hospitalization at admission and discharge, and total hospitalization costs. The recorded diagnoses in the database were validated; for example, the specificity of lung cancer diagnosis was 96.7%, while the sensitivity was 50–80%. The specificity and sensitivity of the recorded procedures exceeded 90% [23, 24].

### Patient selection

We identified inpatients who received cisplatin- or carboplatin-based chemotherapy for lung cancer (ICD-10 code: C34) and olanzapine 2.5 or 5 mg as CINV prophylaxis between January 2016 and March 2021. Prescription of olanzapine at chemotherapy initiation or before was regarded as prophylactic administration of olanzapine. We excluded patients (i) below 18 years old, (ii) with schizophrenia (F20, F22–25, F28, F29), (iii) with diabetes (F10–14) or who received treatment with insulin or oral hypoglycemic agents, or (iv) who received olanzapine for more than two days before chemotherapy or on day ≥ 5 after chemotherapy initiation, assuming that it could be for other purposes such as treatment for general anorexia, cachexia, and psychiatric symptoms [25, 26]. We divided the eligible patients into two groups: patients who were prescribed olanzapine 2.5 mg/day (the 2.5-mg olanzapine group) or olanzapine 5 mg/day (the 5-mg olanzapine group). The initial day of chemotherapy was defined as day 1.

The primary outcome was defined as additional antiemetic drug administration within the overall assessment period (days 2–5) and on each day (2, 3, 4, and 5). We considered the administration as a surrogate for CINV symptoms [9, 27, 28]. According to the antiemetics guidelines and common treatments in Japanese cancer hospitals, metoclopramide, domperidone, lorazepam, alprazolam, haloperidol, chlorpromazine, and prochlorperazine were considered additional antiemetic drugs [29, 30]. The secondary outcomes were dexamethasone use within days 2–5, length of hospital stay, and total hospitalization costs. One US dollar (USD) was equivalent to 110 Japanese yen.

This study was approved by the Institutional Review Board of the University of Tokyo (approval number 3501-5; May 19, 2021). The review board waived the requirement for patient-informed consent because of the use of anonymous data.

### Covariates

We examined the following patients’ characteristics: sex, age, BMI, smoking index (0/1–19/ ≥ 20 pack-years), Charlson comorbidity index [31], Parkinson’s disease, independence in ADL, and cancer stage. The cancer stage was made according to the TNM classification. Age was categorized into four groups: < 64, 65–74, 75–84, and ≥ 85 years. There were four BMI groups: underweight, < 18.5; normal weight, 18.5–24.9; overweight, 25.0–29.9; and obese, ≥ 30 kg/m^2^. Comorbidities were assessed using the Charlson comorbidity index and categorized into 2, 3, 4, or ≥ 5. We also categorized treatment history into radiotherapy, chemotherapy regimen (Supplementary Table 1), antiemetic regimen according to the guidelines of the American Society of Clinical Oncology and National Comprehensive Cancer Network (Supplementary Table 2), use of olanzapine-interacting drugs, number of chemotherapy cycles (one, two, three, or above three), support from a palliative care team, emergency admission, types of hospitals (teaching hospital or not), and fiscal year of admission. Regarding olanzapine-interacting drugs, we included corticosteroids (except dexamethasone), hypnotics (benzodiazepines, non-benzodiazepines, and other hypnotic drugs), barbiturates, selective serotonin reuptake inhibitors, serotonin-norepinephrine reuptake inhibitors, noradrenergic and specific serotonergic antidepressants, tricyclic antidepressants, tetracyclic antidepressants, other antidepressants, multi-acting receptor-targeted antipsychotic drugs (except for olanzapine 2.5 and 5 mg), serotonin-dopamine antagonists, dopamine receptor antagonists (phenothiazines, butyrophenones, and benzamides), anti-parkinsonian drugs (levodopa, levodopa-carbidopa, levodopa-benserazide, ropinirole, pramipexole, and rotigotine), carbamazepine, omeprazole, and rifampicin [16, 17, 32, 33]. We identified patients who received these medications within 7 days before chemotherapy initiation.

### Statistical analysis

We performed a propensity score analysis to adjust for confounding by indication and to compare the outcomes between both groups. A propensity score analysis can effectively adjust for measured confounders and is used to balance patients’ backgrounds in a retrospective study [34]. We conducted propensity score matching at a 1:1 ratio. Propensity scores were calculated with a logistic regression model using the above-mentioned patient and treatment variables as covariates. Each patient who received olanzapine 2.5 mg was matched with a patient who received olanzapine 5 mg using the nearest-neighbor matching method without replacement. The caliper width was ≤ 0.2 of the pooled standard deviation of estimated logits of the propensity score. We calculated standardized differences to examine the balance in baseline covariates of patients between both groups. An absolute standardized difference below 10% denoted a negligible difference between both groups [35]. Continuous and categorical variables were compared using the t test and Chi square test, respectively.

We also performed the four subgroup analyses for the primary outcome. Olanzapine metabolism is reportedly strongly affected by sex, age, and smoking [16, 18–20]; therefore, we evaluated patients in the following subgroups: sex, age (< 65 or ≥ 65 years), and with and without a smoking history. Additionally, we evaluated the data of patients who received only a cisplatin regimen.

Finally, we conducted two sensitivity analyses to confirm the robustness of our results. First, we only included patients who were using three antiemetic drugs (a 5-HT3 receptor antagonist, an NK1 receptor antagonist, and dexamethasone). Second, we conducted an instrumental variable analysis to address unmeasured confounders. Facility proportion of annual 2.5 mg olanzapine usage was an instrumental variable because facility treatment proportion is the best-known instrumental variable type [36, 37]. We used a two-stage residual inclusion estimation framework with robust standard errors, using background patient and treatment characteristics as covariates [38]. An F-statistics of > 10 indicated that our instrumental variable was highly correlated with treatment using additional antiemetic drugs. The first stage was a generalized linear model adjusted for the patient and treatment backgrounds. This model was used to measure the association between the facility proportion of annual olanzapine 2.5 mg usage and our instrument. From this model, we determined the raw residual for each patient by calculating the difference between the model-predicted probability of facility proportion of annual olanzapine 2.5 mg usage and olanzapine 2.5 mg administration. The residuals were then included as an additional covariate in the second-stage model. In the second-stage binomial regression model adjusted for patient and treatment backgrounds used in the first stage and the residual calculated in the first stage, we estimated the association between olanzapine 2.5 or 5 mg administration and additional antiemetic drugs administration. We then calculated a risk difference for the primary outcome (i.e., additional antiemetic drug administration within the overall assessment period) in the olanzapine 2.5 mg group and compared it with that of the olanzapine 5 mg group. To demonstrate the quasi-randomization of treatment assignments by the instrumental variable, we described the patients’ characteristics according to the mean value of the instrumental variable. All analyses were performed using Stata/SE 17.0 software (StataCorp, College Station, TX, USA). Continuous variables are presented as median and interquartile range (IQR). Categorical variables are expressed as numbers and percentages. All reported p values are two-sided, and p < 0.05 was considered statically significant.

## Results

**Fig.1:**
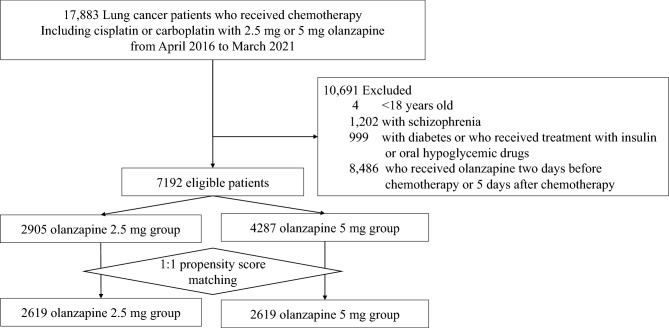
Patient flow chart

We identified 17,883 eligible patients. We excluded 10,691 patients who met the exclusion criteria: (i) four patients were < 18 years old; (ii) 1202 patients had schizophrenia; (iii) 999 had diabetes or used hypoglycemic drugs; and (iv) 8486 received olanzapine two days before chemotherapy or 5 days after chemotherapy. Of the 7192 included patients, 2905 (40%) and 4287 (60%) patients comprised the olanzapine 2.5 and 5 mg groups, respectively (Fig. 1). After 1:1 propensity score matching, each group included 2619 patients; the background characteristics were comparable between the two groups.

Tables 1 and 2 show the background characteristics before and after propensity score matching, respectively. Before the propensity score matching, patients in the olanzapine 2.5 mg group were more likely to be female, older, have a lower BMI, a higher comorbidity index, lower smoking index, and to have received multiple chemotherapy sessions than those in the olanzapine 5 mg group. These backgrounds were well balanced after propensity score matching.

**Table 1:** Characteristics of all the patients and 1:1 propensity score-matched patients

	All patients					1:1 propensity score-matched patients				
	2.5 mg		5 mg		^b^ASD	2.5 mg		5 mg		ASD
	^a^*n* = 2905		*n* = 4287		(%)	*n* = 2619		*n* = 2619		(%)
Male	1712	(59)	2687	(63)	7.7	1558	(59)	1568	(60)	0.8
Age category, years										
18–64	1259	(43)	1871	(44)	0.6	1161	(44)	1157	(44)	0.3
65–74	1303	(45)	2006	(47)	3.9	1194	(46)	1188	(45)	0.5
75–84	328	(11)	405	(9.4)	6.1	263	(10)	269	(10)	0.8
≥ 85	15	(0.5)	5	(0.1)	7.1	1	(0.0)	1	(0.0)	0.0
Body mass index, kg/m^2^										
< 18.5	427	(15)	617	(14)	0.9	392	(15)	391	(15)	0.1
18.5–21.9	1052	(36)	1613	(38)	2.9	941	(36)	955	(36)	1.1
22.0–24.9	931	(32)	1310	(31)	3.2	826	(32)	827	(32)	0.1
25.0–29.9	418	(14)	672	(16)	3.6	402	(15)	395	(15)	0.7
≥ 30	62	(2.1)	69	(1.6)	3.9	53	(2.0)	48	(1.8)	1.4
Missing data	15	(0.9)	6	(0.1)	10.4	5	(0.2)	3	(0.1)	2.0
Smoking index, pack-years										
0	903	(31)	1162	(27)	8.8	837	(32)	832	(32)	0.4
1–19	314	(11)	459	(11)	0.3	301	(11)	307	(12)	0.7
≥ 20	1538	(53)	2432	(57)	7.6	1481	(57)	1480	(57)	0.1
Charlson comorbidity index										
2	1575	(54)	2384	(56)	2.8	1427	(54)	1444	(55)	1.3
3	314	(11)	591	(14)	9.1	282	(11)	284	(11)	0.2
4	112	(3.9)	137	(3.2)	3.6	103	(3.9)	98	(3.7)	0.8
≥ 5	904	(31)	1175	(27)	8.2	808	(31)	793	(30)	1.2
Parkinson's disease	3	(0.1)	1	(0.0)	3.2	1	(0.0)	1	(0.0)	0.0
Independence in^c^ADL	2,628	(90)	3945	(92)	5.5	2388	(91)	2394	(91)	0.8
Cancer stage										
I	258	(8.9)	418	(10)	3.0	235	(9.0)	239	(9.1)	0.5
II	310	(18)	441	(10)	1.3	292	(11)	292	(11)	0.0
III	665	(23)	1080	(25)	5.4	602	(23)	589	(22)	1.2
IV	984	(34)	1488	(35)	1.8	894	(34)	887	(34)	0.6
Missing data	688	(24)	860	(20)	8.8	596	(23)	612	(23)	1.5

^a^Data are presented as *n* (%)

*ASD* absolute standardized difference, *ADL* activities of daily living

An ASD ≤ 10% denotes a negligible difference between the two groups

**Table 2:** Treatment characteristics of all the patients and 1:1 propensity score-matched patients

	All patients					1:1 propensity score-matched patients				
	2.5 mg		5 mg		ASD	2.5 mg		5 mg		ASD
	*n*^a^ = 2905		*n* = 4287		(%)	*n* = 2619		*n* = 2619		(%)
Radiotherapy	281	(10)	369	(8.6)	3.7	243	(9.3)	242	(9.2)	0.1
Chemotherapy regimen										
Cisplatin regimen	1,881	(65)	2804	(65)	1.4	1,726	(66)	1,741	(66)	1.2
Carboplatin regimen	1,027	(35)	1489	(35)	1.3	896	(34)	880	(34)	1.3
Antimetabolites	1,123	(39)	1595	(37)	3.0	1,009	(39)	997	(38)	0.9
Molecular-targeted agents	320	(11)	651	(15)	12.4	298	(11)	277	(11)	2.6
Microtubule-targeted agents	932	(32)	1411	(33)	1.8	842	(32)	846	(32)	0.3
Topoisomerase-1 inhibitors	133	(4.6)	304	(7.1)	10.7	130	(5.0)	131	(5.0)	0.2
Topoisomerase-2 inhibitors	693	(24)	957	(22)	3.6	619	(24)	626	(24)	0.6
Immune checkpoint inhibitor	823	(28)	1248	(29)	1.7	735	(28)	726	(28)	0.8
Antiemetic regimen										
NK1 receptor antagonist	2,639	(91)	3924	(92)	2.4	2,378	(91)	2,386	(91)	1.1
5-HT3 receptor antagonist	2,818	(97)	4084	(95)	9.0	2,539	(97)	2,531	(97)	1.7
Dexamethasone	2,898	(100)	4274	(100)	1.2	2,613	(100)	2,613	(100)	0.0
Olanzapine-interacting drugs use										
Steroid (except dexamethasone)	314	(11)	434	(10)	2.2	260	(10)	262	(10)	0.3
Benzodiazepine	470	(16)	695	(16)	0.1	426	(16)	428	(16)	0.2
Non-benzodiazepine	293	(10)	363	(8.5)	5.6	246	(9.4)	254	(9.7)	1.0
Other hypnotic agents	174	(6.0)	152	(3.5)	11.5	124	(4.7)	123	(4.7)	0.2
Barbiturates	0	(0.0)	1	(0.0)	2.2	0	(0.0)	0	(0.0)	N.A
SSRI	11	(0.4)	23	(0.5)	2.3	11	(0.4)	10	(0.4)	0.6
SNRI	23	(0.8)	32	(0.7)	0.5	20	(0.8)	24	(0.9)	1.7
NaSSA	14	(0.5)	18	(0.4)	0.9	12	(0.5)	14	(0.5)	1.1
Tricyclic antidepressants	4	(0.1)	7	(0.2)	0.7	4	(0.2)	4	(0.2)	0.0
Tetracyclic antidepressants	2	(0.1)	1	(0.0)	2.1	0	(0.0)	1	(0.0)	2.8
Other antidepressants	0	(0.0)	1	(0.0)	2.2	0	(0.0)	0	(0.0)	N.A
MARTA	13	(0.4)	6	(0.1)	5.7	5	(0.2)	5	(0.2)	0.0
Serotonin-dopamine antagonist	10	(0.3)	28	(0.7)	4.4	9	(0.3)	10	(0.4)	0.6
Dopamine partial agonist	2	(0.1)	4	(0.1)	0.9	2	(0.1)	2	(0.1)	0.0
Dopamine receptor antagonists										
Phenothiazines	213	(7.3)	275	(6.4)	3.6	187	(7.1)	204	(7.8)	2.5
Butyrophenones	7	(0.2)	7	(0.2)	1.7	3	(0.1)	6	(0.2)	2.8
Benzamides	0	(0.0)	0	(0.0)	N.A	0	(0.0)	0	(0.0)	N.A
Antiparkinsonian drugs	3	(0.1)	5	(0.1)	0.4	0	(0.0)	0	(0.0)	N.A
Carbamazepine	10	(0.3)	11	(0.3)	1.6	7	(0.3)	10	(0.4)	2.0
Omeprazole	13	(0.4)	11	(0.3)	3.2	10	(0.4)	8	(0.3)	1.3
Rifampicin	0	(0.0)	7	(0.2)	5.7	0	(0.0)	0	(0.0)	N.A
Number of Chemotherapy cycles										
1	215	(7.4)	457	(11)	11.4	201	(7.7)	191	(7.3)	1.5
2	524	(18)	755	(18)	1.1	474	(18)	483	(18)	0.9
3	621	(21)	868	(20)	2.8	568	(22)	590	(23)	2.0
≥ 4	1,545	(53)	2207	(51)	3.4	1,376	(53)	1,355	(52)	1.6
Support from a palliative-care team	88	(3.0)	111	(2.6)	2.7	71	(2.7)	68	(2.6)	0.7
Emergency admission	34	(1.2)	36	(1.3)	1.5	26	(1.0)	26	(1.0)	0.0
Teaching hospital	738	(25)	1041	(24)	2.6	647	(25)	675	(26)	2.5
Fiscal year of chemotherapy										
2016	194	(6.7)	382	(8.9)	8.3	180	(6.9)	176	(6.7)	0.6
2017	330	(11)	567	(13)	5.7	302	(12)	308	(12)	0.7
2018	759	(26)	1024	(24)	5.2	675	(26)	663	(25)	1.1
2019	753	(26)	1000	(23)	6.0	673	(26)	671	(26)	0.2
2020	869	(30)	1314	(31)	1.6	789	(30)	801	(31)	1.0

^a^Data are presented as *n* (%)

*ASD* absolute standardized difference, *SSRI* selective serotonin reuptake inhibitor, *SNRI* serotonin-norepinephrine reuptake inhibitor, *NaSSA* noradrenergic and specific serotonergic antidepressants, *MARTA* multi-acting receptor-targeted antipsychotic

An ASD ≤ 10% denotes a negligible difference between the two groups

Table 3 shows the outcomes of all the patients and those of the 1:1 propensity score-matched patients. Before matching, the olanzapine 2.5 mg group had a higher proportion of patients with primary outcomes within the overall assessment period (days 2–5) (36% vs. 28%). The propensity score-matched analysis showed that the olanzapine 2.5 mg group was associated with a higher proportion of additional antiemetic drug administration within the overall assessment period (36% vs. 31%; difference, 6.2% [95% confidence interval, CI 3.7 to 8.7]) but a lower proportion of dexamethasone on days 2 (38% vs. 40%, difference, -1.9% [95% CI -4.5 to 0.7]), 3 (13% vs. 16%; difference, -3.3% [95% CI -5.2 to -1.4]), and 4 (13% vs. 15%; difference, -2.1% [95% CI -3.9 to -0.2], when compared to the olanzapine 5 mg group. The median length of hospital stay was not significantly different between both groups (8 vs. 8 days; difference, 0 days [95% CI -0.5 to 0.7]). Total hospitalization costs were not significantly different between both groups (5061 vs. 5160 USD; difference, -108 USD [95% CI -396 to 180]).

**Table 3:** Comparisons of outcomes before and after propensity score matching

	All patients				1:1 propensity score-matched patients					
	2.5 mg		5 mg		2.5 mg		5 mg		Risk difference	95% CI
	*n* = 2905		*n* = 4287		*n* = 2619		*n* = 2619			
	*n*	(%)	*n*	(%)	*n*	(%)	*n*	(%)	(%)	(%)
Additional antiemetic drug administration										
Days 2–5 after chemotherapy	1046	(36)	1213	(28)	941	(36)	811	(31)	6.2	3.7 to 8.7
Day 2 after chemotherapy	579	(20)	657	(15)	526	(20)	403	(15)	4.7	2.6 to 6.8
Day 3 after chemotherapy	514	(18)	542	(13)	459	(18)	341	(13)	4.5	2.6 to 6.5
Day 4 after chemotherapy	428	(15)	480	(11)	382	(15)	316	(12)	2.5	0.7 to 4.4
Day 5 after chemotherapy	235	(8.1)	340	(7.9)	210	(8.0)	222	(8.5)	-0.5	-2.0 to 1.0
Day 6 after chemotherapy	222	(7.6)	289	(6.7)	199	(7.6)	186	(7.1)	0.5	-0.9 to 2.0
Day 7 after chemotherapy	217	(7.5)	243	(5.7)	192	(7.3)	154	(5.9)	1.5	0.0 to 2.8
Dexamethasone administration										
Day 2 after chemotherapy	1089	(37)	1697	(40)	988	(38)	1038	(40)	-1.9	-4.5 to 0.7
Day 3 after chemotherapy	374	(13)	679	(16)	332	(13)	418	(16)	-3.3	-5.2 to -1.4
Day 4 after chemotherapy	383	(13)	601	(14)	337	(13)	391	(15)	-2.1	-3.9 to -0.2
Day 5 after chemotherapy	64	(2.2)	96	(2.2)	56	(2.1)	62	(2.4)	-0.2	-1.0 to 0.01
	Median	IQR	Median	IQR	Median	IQR	Median	IQR	Difference	95% CI
Length of hospital stay, days	8	(4–14)	8	(4–12)	8	(4–14)	8	(4–13)	0	-0.5 to 0.7
Total hospitalization costs, US dollars	5106	(2658–8871)	5153	(2874–8751)	5061	(2644–8759)	5160	(2913–8759)	-108	-396 to 180

*IQR* interquartile range, *CI* confidence interaval

The subgroup analyses showed similar results to those of analyses of the 1:1 propensity score-matched cohort (Tables 4, 5). In the subgroups of male sex, age ≥ 65 years, and smoker status, the proportion of patients who received additional antiemetics was significantly higher in the olanzapine 2.5 mg group than in the olanzapine 5 mg group. No significant difference was observed in the female sex and age < 65 years subgroups. The patients’ background characteristics categorized according to the mean value of the instrumental variable are shown in Supplemental Tables 3 and 4. The olanzapine 2.5 mg group was significantly associated with higher use of additional antiemetic drugs within the overall assessment period (risk difference, 0.076 [95% CI 0.055 to 0.10]). Sensitivity analysis for patients using three antiemetic drugs demonstrated consistent results (Supplemental Table 5).

**Table 4:** Subgroup analyses by sex, age, and smoking history using 1:1 propensity score-matched analyses to compare additional antiemetic drug administration between the 2.5 mg and 5 mg olanzapine group

Subgroup analysis	1:1 propensity score-matched patients											
Stratified by sex	Male						Female					
	2.5 mg		5 mg		Risk difference	95% CI	2.5 mg		5 mg		Risk difference	95% CI
	^a^*n* = 1515		*n* = 1515				*n* = 1013		*n* = 1013			
Day 2–5 after chemotherapy	586	(39)	456	(30)	8.4	5.1 to 11.7	326	(32)	298	(29)	2.8	-1.3 to 6.8
Day 2 after chemotherapy	344	(23)	262	(17)	5.4	2.6 to 8.3	161	(16)	128	(13)	3.3	0.2 to 6.3
Day 3 after chemotherapy	282	(19)	214	(14)	4.5	1.9 to 7.1	156	(15)	127	(13)	2.9	-0.2 to 5.9
Day 4 after chemotherapy	220	(15)	173	(11)	3.1	0.7 to 5.5	148	(15)	129	(13)	1.9	-1.1 to 4.9
Day 5 after chemotherapy	124	(8.2)	111	(7.3)	0.9	-1.0 to 2.8	78	(7.7)	99	(10)	-2.1	-4.6 to 0.4

Stratified by age	Age < 65 years						Age ≥ 65 years					
	2.5 mg		5 mg		Risk difference	95% CI	2.5 mg		5 mg		Risk difference	95% CI
	*n* = 1106		*n* = 1106				*n* = 1417		*n* = 1417			
Day 2–5 after chemotherapy	435	(39)	377	(34)	5.2	1.2 to 9.2	464	(33)	372	(26)	6.5	3.2 to 9.8
Day 2 after chemotherapy	241	(22)	223	(20)	1.6	-1.8 to 5.0	262	(18)	196	(14)	4.7	2.0 to 7.4
Day 3 after chemotherapy	209	(19)	175	(16)	3.1	-0.1 to 6.2	226	(16)	166	(12)	4.2	1.7 to 6.9
Day 4 after chemotherapy	173	(16)	145	(13)	2.5	-0.4 to 5.5	187	(13)	149	(11)	2.7	0.3 to 5.1
Day 5 after chemotherapy	103	(9.3)	95	(8.6)	0.7	-1.7 to 3.1	99	(7.0)	110	(7.8)	-0.7	-2.7 to 1.2

Stratified by smoking history	Smokers						Never smokers					
	2.5 mg		5 mg		Risk difference	95% CI	2.5 mg		5 mg		Risk difference	95% CI
	*n* = 1929		*n* = 1929				*n* = 778		*n* = 778			
Day 2–5 after chemotherapy	639	(33)	525	(27)	7.9	5.0 to 10.8	264	(34)	237	(30)	3.5	-1.2 to 8.1
Day 2 after chemotherapy	401	(21)	291	(15)	5.7	3.3 to 8.1	147	(19)	121	(16)	3.3	-0.4 to 7.1
Day 3 after chemotherapy	356	(18)	262	(14)	4.9	2.6 to 7.2	127	(16)	97	(12)	3.9	0.4 to 7.3
Day 4 after chemotherapy	288	(15)	242	(13)	2.4	0.2 to 4.6	116	(15)	89	(12)	3.5	0.1 to 6.9
Day 5 after chemotherapy	158	(8.2)	173	(9.0)	-0.8	-2.5 to 1.0	64	(8.2)	74	(10)	-1.3	-4.1 to 1.5

^a^Data are presented as *n* (%). −

*CI* confidence interval

**Table 5:** Subgroup analyses by chemotherapy regimen using 1:1 propensity score-matched analyses to compare additional antiemetic drug administration between the 2.5 mg and 5 mg olanzapine group

Subgroup analysis	1:1 propensity score-matched patients						Carboplatin regimen					
Stratified by chemotherapy regimen	Cisplatin regimen											
	2.5 mg		5 mg		Risk difference	95% CI	2.5 mg		5 mg		Risk difference	95% CI
	*n* = 1668		*n* = 1668				*n* = 807		*n* = 807			
Day 2–5 after chemotherapy	646	(39)	574	(34)	4.3	1.1 to 7.6	240	(30)	179	(22)	7.5	3.3 to 12.8
Day 2 after chemotherapy	366	(22)	324	(19)	2.5	-0.2 to 5.3	127	(16)	73	(9.0)	6.8	3.5 to 10.1
Day 3 after chemotherapy	314	(19)	269	(16)	2.7	0.1 to 5.3	116	(14)	62	(7.7)	6.8	3.6 to 10.0
Day 4 after chemotherapy	271	(16)	228	(14)	2.6	0.2 to 5.0	94	(12)	81	(10)	1.6	-1.4 to 4.7
Day 5 after chemotherapy	145	(8.7)	151	(9.1)	-0.4	-2.3 to 1.6	55	(6.8)	72	(8.9)	-2.1	-4.8 to 0.5

Data are presented as *n* (%)

*CI* confidence interval

## Discussion

In this study, using a nationwide database in Japan, we compared the outcomes of olanzapine 2.5 mg and 5 mg in terms of CINV prophylaxis. Olanzapine 2.5 mg administration was associated with a higher proportion of patients using additional antiemetics than olanzapine 5 mg. However, in the female sex or age < 65 years subgroups, the proportion of use of additional antiemetics in the olanzapine 2.5 mg group was not significantly different to that in the olanzapine 5 mg group. The length of hospital stay was not significantly different between both groups.

The universal healthcare insurance system in Japan began to cover the cost of olanzapine used as CINV prophylaxis in June 2017. The rate of olanzapine administration as CINV prophylaxis in Japan has increased to that in the US [12, 30]. Our study showed that olanzapine 2.5 mg was administered to mainly females, elderly patients, and patients who received chemotherapy multiple times. These differences were adjusted for in our propensity score analyses.

The olanzapine 2.5 mg group needed additional antiemetics more frequently than the 5 mg group, especially within days 2–4. Our results are consistent with those of randomized controlled trials of olanzapine 5 mg used as CINV prophylaxis; these trials recorded similar trends of additional antiemetics administration [9, 27]. Patients who receive HEC are likely to experience severe CINV on days 2–5 [39]. The relationship between plasma concentration of olanzapine and dose is linear; therefore, the efficacy of olanzapine 2.5 mg may be inferior to that of 5 mg in CINV [16]. In this study, the length of hospital stay and total hospitalization costs did not differ between both groups. These outcomes could have been affected by the side effects of olanzapine 5 mg such as sleepiness or somnolence.

A previous study of patients with breast cancer showed the efficacy and safety of olanzapine 2.5 mg for CINV [13]. However, the report included only few patients (n = 45) with breast cancer. Consequently, the patient’s characteristics that may affect the efficacy of olanzapine (e.g., sex, age, smoking history, and olanzapine-interacting drugs use) were not adjusted in the analysis [16, 17]. Additionally, the study did not compare the outcomes between olanzapine 2.5 and 5 mg administration. Contrarily, our study showed the advantageous effectiveness of olanzapine 5 mg over 2.5 mg by adjusting patient characteristics using a nationwide database.

This is the first study to explore the relationship between factors affecting the metabolism of olanzapine and the antiemetic efficacy of varying dosages. A difference was found in the subgroups of male sex and age ≥ 65 years. However, a previous randomized trial showed no difference between olanzapine 5 mg and placebo groups by sex or age [21]. The results in our study differ from those in the previous study. Our study used retrospective data, and nausea was assessed solely based on an antiemetic prescription. Therefore, nausea that required no antiemetics may not have been adequately evaluated. We analyzed the effect of smoking history in subgroup analysis. Some studies also reported that the increased clearance of olanzapine due to smoking needs a few days to disappear after smoking cessation [18, 20, 40].Thus, the proportion of additional antiemetics administration did significantly differ in a subgroup with smokers at admission.

Propensity score analysis has advantages compared with traditional multivariable regression in that it can eliminate measured confounding by balancing the confounders and directly estimate the treatment effect. However, propensity score analysis has a limitation in that it cannot adjust for unmeasured confounders. In the present study, unmeasured confounders (e.g., brain metastasis, dosage of cisplatin or carboplatin, and previous history of nausea) may have influenced the olanzapine doses. We therefore conducted instrumental variable analysis, which can theoretically adjust even for unmeasured confounders. The results of the instrumental variable analysis, which accounted for unmeasured background factors, supported our findings of the main analysis (1:1 propensity score analysis).

This study has several limitations. First, the CINV symptoms could have been underestimated as the database does not contain patient-reported outcomes; we used additional antiemetics administration as an alternative to CINV symptoms. Therefore, we could only investigate patients with severe nausea or vomiting. Previous trials have reported that nausea occurred in 40% of patients who receive olanzapine 5 mg after chemotherapy. In this study, the proportion of patients with additional antiemetics was 22% [9]. However, because the underestimation would have occurred equally in both groups, it would not have skewed our results. Additionally, we could not evaluate outcomes in the outpatient settings after discharge because we only investigated the additional antiemetics administered during hospitalization. However, the underestimation of outcomes was small because patients in this study were hospitalized for approximately one week. Second, we were able to evaluate the prescription but not the actual administration of olanzapine. Patients in the olanzapine 5 mg group may have stopped olanzapine due to the side effects (e.g., sleepiness in the daytime) [9–11]. Nevertheless, the olanzapine 5 mg group received significantly fewer additional antiemetics than the olanzapine 2.5 mg group. Therefore, the results may have not been altered by the lack of information on actual olanzapine administration. Third, we compared between the groups using propensity score-matched analysis. Although we generated 2626 pairs for analysis, we lost approximately 1800 patients. Moreover, propensity score analysis is not able to adjust for unmeasured confoundings. However, we adjusted for more than 40 measured confoundings including type of drugs, chemotherapy regimen, and antiemetics regimens. There were few unmeasured confoundings in our study. Fourth, although the number of patients using three antiemetic drugs was small, the results in the sensitivity analysis, which only included patients who received all three antiemetic drugs, were similar to those in the main results.

## Conclusion

Olanzapine 2.5 mg administration for CINV prophylaxis was significantly associated with additional antiemetics administration. However, among female patients or patients aged < 65 years, the outcomes of olanzapine 2.5 mg was not significantly different from those in the olanzapine 5 mg group—in terms of additional antiemetics administration.

## Supplementary Information

Below is the link to the electronic supplementary material.

Supplementary file1 (DOCX 49 KB)
