# Supplementary material for: Comparison of olanzapine 2.5 mg and 5 mg in the prevention of chemotherapy-induced nausea and vomiting: a Japanese nationwide database study
Source: Int J Clin Oncol. 2024 Aug 18;29(11):1762–73. doi: 10.1007/s10147-024-02603-2 (PMC11511693; doi:10.1007/s10147-024-02603-2)
Supplement: Supplementary file 1 — Supplementary file1 (DOCX 49 KB) [file 10147_2024_2603_MOESM1_ESM.docx]

Comparison of olanzapine 2.5 mg and 5 mg in the prevention of chemotherapy-induced nausea and vomiting:

a Japanese nationwide database study

Hiroe Suzuki-Chiba, Takaaki Konishi, Shotaro Aso, Kanako Makito,

Hiroki Matsui, Taisuke Jo, Kiyohide Fushimi , Hideo Yasunaga

Correspondence to

Hiroe Suzuki-Chiba

E-mail: hiroechiba@m.u-tokyo.ac.jp

Supplemental Table 1. Chemotherapy regimen

Supplemental Table 2. Evaluated antiemetics regimen

Supplemental Table 3. Patients’ characteristics according to the mean value of an instrumental variable

Supplemental Table 4. Patients’ treatment characteristics according to the mean value of an instrumental variable

Supplemental Table 5. Comparisons of outcomes before and after propensity score matching in patients who received three antiemetic drugs

**Supplemental Table 1.** **Chemotherapy regimen**

| **Category** | **Chemotherapy** |
| --- | --- |
| Cisplatin regimen | Cisplatin |
| Carboplatin regimen | Carboplatin |
| Antimetabolites | Pemetrexed  Pemetrexed disodium  Pemetrexed disodium heptahydrate  Gimeracil-oteracil potassium-tegafur  Gemcitabine  Gemcitabine hydrochloride |
| Molecular-targeted agents | Bevacizumab |
| Microtubule-targeted agents | Paclitaxel  Paclitaxel albumin  Docetaxel  Vinorelbine tartrate  Vincristine |
| Topoisomerase-1 inhibitors | Irinotecan |
| Topoisomerase-2 inhibitors | Etoposide |
| Immune checkpoint inhibitor | Pembrolizumab  Nivolumab  Atezolizumab  Durvalumab  Ipilimumab |

**Supplemental Table 2. Evaluated antiemetics regimen**

| **Antiemetic category** | **Drugs** |
| --- | --- |
| NK1-receptor antagonists | Aprepitant  Fosaprepitant meglumine |
| 5-HT3 receptor | Granisetron hydrochloride  Indisetron hydrochloride  Azasetron hydrochloride  Ondansetron hydrochloride  Ramosetron hydrochloride  Palonosetron hydrochloride |
| Dexamethasone | Dexamethasone |

Abbreviations: NK1, neurokinin 1; 5-HT3, 5-hydroxytryptamine-3

**Supplemental Table 3.** **Patients’ characteristics according to the mean value of an instrumental variable**

|  | Facility rate of annual 2.5 mg olanzapine usage | | | | |
| --- | --- | --- | --- | --- | --- |
|  | ratio <0.41 | | ratio ≥0.41 | | ASD |
|  | n=3916 | | n=3276 | | (%) |
| olanzapine 2.5 mg | 225 | (5.7) | 2680 | (82) | 238.8 |
| Male | 2,457 | (63) | 1,942 | (59) | 7.1 |
| Age category, years |  |  |  |  |  |
| 18–64 | 1,721 | (44) | 1,409 | (43) | 1.9 |
| 65–74 | 1,804 | (46) | 1,505 | (46) | 0.3 |
| 75–84 | 388 | (10) | 345 | (11) | 2.1 |
| ≥85 | 3 | (0.1) | 17 | (0.5) | 8.1 |
| Body mass index, kg/m^2^ |  |  |  |  |  |
| <18.5 | 583 | (15) | 461 | (14) | 2.3 |
| 18.5–21.9 | 1,446 | (37) | 1,219 | (37) | 0.6 |
| 22.0–24.9 | 1,201 | (31) | 1,040 | (32) | 2.3 |
| 25.0–29.9 | 615 | (16) | 475 | (14) | 3.4 |
| ≥30 | 64 | (1.6) | 67 | (2.0) | 3.1 |
| Missing data | 7 | (0.2) | 14 | (0.4) | 4.5 |
| Smoking index, pack-years |  |  |  |  |  |
| 0 | 1,104 | (28) | 961 | (29) | 2.5 |
| 1–19 | 417 | (11) | 356 | (11) | 0.7 |
| ≥20 | 2,183 | (56) | 1,787 | (55) | 2.4 |
| Charlson comorbidity index |  |  |  |  |  |
| 2 | 2,180 | (56) | 1,779 | (54) | 2.7 |
| 3 | 516 | (13) | 389 | (12) | 3.9 |
| 4 | 125 | (3.2) | 124 | (3.8) | 3.2 |
| ≥5 | 1,095 | (28) | 984 | (30) | 4.6 |
| Parkinson's disease | 0 | (0.0) | 4 | (0.1) | 4.9 |
| Independence in ADL | 3,585 | (92) | 2,988 | (91) | 1.2 |
| Cancer stage |  |  |  |  |  |
| Ⅰ | 378 | (9.7) | 298 | (9.1) | 1.9 |
| Ⅱ | 403 | (10) | 348 | (11) | 1.1 |
| Ⅲ | 952 | (24) | 793 | (24) | 0.2 |
| Ⅳ | 1,398 | (36) | 1,074 | (33) | 6.1 |
| Missing data | 785 | (20) | 763 | (23) | 7.9 |

Data are presented as *n* (%).

An ASD ≤10% denotes a negligible difference between the two groups. The F-statistic was 1848.

Abbreviations: ASD, absolute standardized difference; ADL: activities of daily living

**Supplemental Table 4.** **Patients’ treatment characteristics according to the mean value of an instrumental variable**

|  | Facility rate of annual 2.5 mg olanzapine usage | | | | |
| --- | --- | --- | --- | --- | --- |
|  | ratio <0.41 | | ratio ≥0.41 | | ASD |
|  | n=3916 | | n=3276 | | (%) |
| Radiotherapy | 312 | (8.0) | 338 | (10) | 8.2 |
| Chemotherapy regimen |  |  |  |  |  |
| Cisplatin regimen | 2,509 | (64) | 2,176 | (66) | 4.9 |
| Carboplatin regimen | 1,411 | (36) | 1,105 | (34) | 4.8 |
| Antimetabolites | 1,477 | (38) | 1,241 | (38) | 0.3 |
| Molecular-targeted agents | 635 | (16) | 336 | (10) | 17.7 |
| Microtubule-targeted agents | 1,287 | (33) | 1,056 | (32) | 1.3 |
| Topoisomerase-1 inhibitors | 281 | (7.2) | 156 | (4.8) | 10.2 |
| Topoisomerase-2 inhibitors | 855 | (22) | 795 | (24) | 5.8 |
| Immune checkpoint inhibitor | 1,194 | (30) | 877 | (27) | 8.2 |
| Antiemetic regimen |  |  |  |  |  |
| NK1 receptor antagonist | 3,592 | (92) | 2,971 | (91) | 3.7 |
| 5-HT3 receptor antagonist | 3,718 | (95) | 3,184 | (97) | 11.6 |
| Dexamethasone | 3,907 | (100) | 3,265 | (100) | 2.0 |
| Drugs for daily use |  |  |  |  |  |
| Steroids (except dexamethasone) | 409 | (10) | 339 | (10) | 0.3 |
| Benzodiazepine | 619 | (16) | 546 | (17) | 2.3 |
| Non-benzodiazepine | 335 | (8.6) | 321 | (9.8) | 4.3 |
| Other hypnotic agents | 133 | (3.4) | 193 | (5.9) | 11.9 |
| Barbiturates | 1 | (0.0) | 0 | (00) | 2.3 |
| SSRI | 20 | (0.5) | 14 | (0.4) | 1.2 |
| SNRI | 29 | (0.7) | 26 | (0.8) | 0.6 |
| NaSSA | 18 | (0.5) | 14 | (0.4) | 0.5 |
| Tricyclic antidepressants | 7 | (0.2) | 4 | (0.1) | 1.5 |
| Tetracyclic antidepressants | 1 | (0.0) | 2 | (0.1) | 1.7 |
| Other antidepressants | 1 | (0.0) | 0 | (0.0) | 2.3 |
| MARTA | 7 | (0.2) | 12 | (0.4) | 3.6 |
| Serotonin-dopamine antagonist | 19 | (0.5) | 19 | (0.6) | 1.3 |
| Dopamine partial agonist | 3 | (0.1) | 3 | (0.1) | 0.5 |
| Dopamine receptor antagonists |  |  |  |  |  |
| Phenothiazines | 236 | (6.0) | 252 | (7.7) | 6.6 |
| Butyrophenones | 9 | (0.2) | 5 | (0.2) | 1.8 |
| Benzamides | 0 | (0.0) | 0 | (0.0) | N.A. |
| Antiparkinsonian drugs | 2 | (0.1) | 6 | (0.2) | 3.9 |
| Carbamazepine | 10 | (0.3) | 11 | (0.3) | 1.5 |
| Omeprazole | 10 | (0.3) | 14 | (0.4) | 2.9 |
| Rifampicin | 7 | (0.2) | 0 | (0.0) | 6.0 |
| Number of Chemotherapy cycles |  |  |  |  |  |
| 1 | 418 | (11) | 262 | (8.0) | 9.2 |
| 2 | 654 | (17) | 625 | (19) | 6.2 |
| 3 | 784 | (20) | 705 | (22) | 3.7 |
| ≥4 | 2,068 | (53) | 1,684 | (51) | 2.8 |
| Support from a palliative-care team | 99 | (2.5) | 100 | (3.1) | 3.2 |
| Emergency admission | 33 | (0.8) | 37 | (1.1) | 2.9 |
| Teaching hospital | 891 | (23) | 888 | (27) | 10.1 |
| Fiscal year of chemotherapy |  |  |  |  |  |
| 2016 | 351 | (9.0) | 225 | (6.9) | 7.8 |
| 2017 | 525 | (13) | 372 | (11) | 6.2 |
| 2018 | 966 | (25) | 817 | (25) | 0.6 |
| 2019 | 923 | (24) | 830 | (25) | 4.1 |
| 2020 | 1151 | (29) | 1032 | (32) | 4.6 |
| Additional antiemetics on days 2–5 | 1103 | (28) | 1156 | (35) | 15.3 |

Data are presented as *n* (%).

Abbreviations: ASD, absolute standardized difference; SSRI, selective serotonin reuptake inhibitor; SNRI, serotonin-norepinephrine reuptake inhibitor; NaSSA, noradrenergic and specific serotonergic antidepressants; MARTA, multi-acting receptor-targeted antipsychotic

An ASD of ≤10% denotes a negligible difference between the two groups.

**Supplemental Table 5.** **Comparisons of outcomes before and after propensity score matching in patients who received three antiemetic drugs**

|  | All patients | | | |  | 1:1 propensity score-matched patients | | | | | |
| --- | --- | --- | --- | --- | --- | --- | --- | --- | --- | --- | --- |
|  | 2.5 mg | | 5 mg | |  | 2.5 mg | | 5 mg | | Risk  difference | 95% CI |
|  | n=2556 | | n=3721 | |  | n=2294 | | n=2294 | |  |  |
|  | n | (%) | n | (%) |  | n | (%) | n | (%) | (%) | (%) |
| Additional antiemetic drug administration |  | |  | |  |  | |  | |  |  |
| Days 2–5 after chemotherapy | 931 | (36) | 1100 | (30) |  | 825 | (36) | 701 | (31) | 5.4 | 2.7 to 8.1 |
| Day 2 after chemotherapy | 515 | (20) | 605 | (16) |  | 464 | (20) | 372 | (16) | 4.0 | 1.8 to 6.2 |
| Day 3 after chemotherapy | 457 | (18) | 496 | (13) |  | 404 | (18) | 321 | (14) | 3.6 | 1.5 to 5.7 |
| Day 4 after chemotherapy | 384 | (15) | 437 | (12) |  | 337 | (15) | 275 | (12) | 2.7 | 0.7 to 4.7 |
| Day 5 after chemotherapy | 209 | (8.2) | 314 | (8.4) |  | 182 | (7.9) | 193 | (8.4) | -0.5 | -2.1 to 1.1 |
| Day 6 after chemotherapy | 198 | (7.7) | 260 | (7.0) |  | 175 | (7.6) | 165 | (7.2) | 0.4 | -1.1 to 2.0 |
| Day 7 after chemotherapy | 196 | (7.7) | 219 | (5.9) |  | 173 | (7.5) | 136 | (5.9) | 1.6 | 0.2 to 3.1 |

Abbreviation: IQR, interquartile range; CI, confidence interaval
